# Saliency of breast lesions in breast cancer detection using artificial intelligence

**DOI:** 10.1038/s41598-023-46921-3

**Published:** 2023-11-23

**Authors:** Said Pertuz, David Ortega, Érika Suarez, William Cancino, Gerson Africano, Irina Rinta-Kiikka, Otso Arponen, Sara Paris, Alfonso Lozano

**Affiliations:** 1 Escuela de Ingenierías Eléctrica Electrónica y de Telecomunicaciones, Universidad Industrial de Santander, Bucaramanga, Colombia; 2 Faculty of Medicine and Health Technology, Tampere University, Tampere, Finland; 3 Department of Radiology, Tampere University Hospital, Tampere, Finland; 4 Departamento de Imágenes Diagnósticas, Universidad Nacional de Colombia, Bogotá, Colombia

**Keywords:** Breast cancer, Diagnostic markers

## Abstract

The analysis of mammograms using artificial intelligence (AI) has shown great potential for assisting breast cancer screening. We use saliency maps to study the role of breast lesions in the decision-making process of AI systems for breast cancer detection in screening mammograms. We retrospectively collected mammograms from 191 women with screen-detected breast cancer and 191 healthy controls matched by age and mammographic system. Two radiologists manually segmented the breast lesions in the mammograms from CC and MLO views. We estimated the detection performance of four deep learning-based AI systems using the area under the ROC curve (AUC) with a 95% confidence interval (CI). We used automatic thresholding on saliency maps from the AI systems to identify the areas of interest on the mammograms. Finally, we measured the overlap between these areas of interest and the segmented breast lesions using Dice’s similarity coefficient (DSC). The detection performance of the AI systems ranged from low to moderate (AUCs from 0.525 to 0.694). The overlap between the areas of interest and the breast lesions was low for all the studied methods (median DSC from 4.2% to 38.0%). The AI system with the highest cancer detection performance (AUC = 0.694, CI 0.662–0.726) showed the lowest overlap (DSC = 4.2%) with breast lesions. The areas of interest found by saliency analysis of the AI systems showed poor overlap with breast lesions. These results suggest that AI systems with the highest performance do not solely rely on localized breast lesions for their decision-making in cancer detection; rather, they incorporate information from large image regions. This work contributes to the understanding of the role of breast lesions in cancer detection using AI.

## Introduction

In recent years, artificial intelligence (AI) systems have shown great potential in the analysis of screening mammograms for breast cancer detection^1^. One of the main difficulties for their adoption in clinical practice is the limited understanding of how these systems make their decisions^2^. Specifically, AI systems often involve complex computational layers with millions of parameters, which hinders the interpretation of the system’s output by humans. This difficulty is often referred to as the “black box” problem of AI^3,4^.

To overcome this problem, explainable AI (XAI) has been developed to provide methods for understanding the decision-making of AI systems. A recent survey found that 37% of diagnostic test studies in radiology incorporate some form of XAI^5^. The main approaches to XAI can be classified depending on their use of visualization, semantics, or counterexamples. Visualization methods, also known as visual explanation or saliency mapping methods, are the most common form of XAI^6^. *Saliency mapping* is used to generate “heatmaps” that highlight the areas in medical images that most influence the prediction of the AI system^7^. Semantic methods, also known as textual explanation methods, provide text descriptions. Counterexamples or example-based explanation methods provide examples related to the data under analysis. A comparison of these methods of explanation is beyond the scope of this work. For a detailed review of XAI methods, we refer the reader to (Reyes et al.^3^) and (Van der Velden et al.^6^).

In this work, we use saliency analysis to compare the overlap between areas of interest from AI systems against the localization of breast lesions segmented by expert radiologists in screening mammograms. In the analysis of mammograms, the presence of suspicious regions or lesions plays a pivotal role in the decision-making process of human readers^8^. In turn, because AI-based systems are trained on clinical findings, it is reasonable to assume that the lesions found in the mammograms will also play a fundamental role in AI-based analysis, which should be reflected in saliency maps^9^. Our hypothesis is that the areas of interest found by saliency analysis of AI systems should be associated with the localization of lesions. Thus, we expect that, for an AI-based method to perform well in breast cancer detection, saliency analysis should reveal a greater attention given to areas close to lesions. This work aims to contribute to understanding how AI systems detect malignancies by studying the role of the location of lesions in the decision-making process of these systems.

## Materials and methods

### Imaging data and lesion segmentation

To assess the detection performance of AI systems, we adopted a case–control design approach and matched cases and controls by age, screening year, and the mammographic system. We retrospectively collected mammograms from the breast cancer screening population of Tampere University Hospital. In Finland, women aged between 50 and 69 years are invited to a every two years mammographic breast cancer screening. Between 2015 and 2017, over 30,000 women were screened, and we identified 277 women with breast cancer during that period. We assessed all 277 women for the following inclusion criteria: (1) no known history of previously detected breast malignancies or previous invasive operations in the field of view (e.g., lumpectomy, mastectomy, breast implant, coiling, pacemaker), as these were hypothesized to have an impact on breast parenchyma and (2) screening-detected unilateral non-invasive or invasive cancer (i.e., no reported breast-cancer related symptoms, as they would require further management irrespective of the mammographic result). The patients who did not meet the inclusion criteria were excluded (*N* = 86). We identified 191 patients diagnosed with asymptomatic screening-detected, biopsy-proven cancer during the index years (2015 to 2017). All the patients who were included had been diagnosed with unilateral cancer. Corresponding healthy controls were matched by birth years, screening years, and mammographic system. The use of registered data, including mammographic images and patient history, was approved, and the need for informed consent was waived by the Research Chair of the Tampere University Hospital (permission number R18047 and R20603) in compliance with local and national regulations and laws. Because of its retrospective nature, the study was not subject to an ethics review. This retrospective study did not change either the diagnostic decisions or the management of the patients. Of the 382 women included in this study (191 cases plus 191 controls), 228 had been included in previous reports^10,11^. These reports concerned radiomic analysis for assessing the risk of breast cancer in *future* screening rounds, whereas in this work we are interested in breast cancer detection in the *current* screening round. There is therefore no overlap in the imaging data with previous studies.

Imaging data was retrieved from the patient flow management software (Optomed Software, Optomed Ltd, Finland). We used bilateral two-view cranio-caudal (CC) and mediolateral oblique (MLO) full-field digital mammography images (1,582 images from 382 women). Mammograms were acquired using either a MicroDose SI (Philips Healthcare [PH], the Netherlands) or a Senographe Essential (General Electric Medical Systems [GE], USA) mammography system. All images were retrieved and standardized to a resolution of 100 µm/pixel and stored in 16-bit format. The study sample is summarized in Table 1.

**Table 1:** Summary of study sample.

Characteristic	Cases (%) *N* = 143		Controls (%) *N* = 143	
Age				
< 55	30	(21)	30	(21)
55–59	40	(28)	40	(28)
60–64	53	(37)	53	(37)
> 64	20	(14)	20	(14)
Mammographic system				
Philips^a^	31	(22)	31	(22)
GE^b^	112	(78)	112	(78)
Cancer type				
DCIS	25	(17)	–	
Ductal	91	(64)	–	
Lobular	18	(13)	–	
Other	9	(6)	–	

^a^MicroDose SI (Philips Healthcare, the Netherlands).

^b^Senographe Essential (GE Medical Systems, USA).

For the manual delineation of breast lesions, we used software developed in-home and implemented in ImageJ^12^. We considered both views (CC and MLO) from the affected breast in all cases (191 women, 382 images). Lesions were manually segmented by delineating their contours on mammograms. The contour was established by consensus between a radiologist with more than 20 years of experience and a resident radiologist. It was not possible to generate segmentations for 12 images, since the lesions were only conspicuous from one of the mammographic views. As a result, 370 segmentations were considered in our saliency analysis. In this work, the manual contours drawn by the two radiologists are considered the ground truth of the breast lesions.

### Detection performance and saliency analysis

We considered four state-of-the-art AI systems for the detection of breast cancer in screening mammography: the end-to-end deep learning architecture (End2End)^13^, the deep multi-view convolutional neural network (DMV-CNN)^14^, the globally aware multiple instance classifier (GMIC)^9^, and the system based on global-local activation maps (GLAM)^15^. These systems generate a prediction score associated with the presence of malignancies. Because we were interested in analyzing the interaction between the presence of breast lesions and saliency, all systems were set up to generate a prediction score on a per-image basis. In this study, the cancer detection task involves predicting whether there is a malignant lesion in the mammogram. Thus, the output of the systems is analyzed as a binary classification problem. The systems considered in this work are summarized in Table 2. In the table, the test population and AUCs correspond to the information reported by the respective developers of each system at the patient level. Confidence intervals are not included, since they were not reported in the original works.

**Table 2:** Artificial intelligence systems for breast cancer detection.

System	Year	Test population	AUC
GLAM^15^	2021	14,148 Exams from the NYU Breast Cancer Screening dataset v1^16^. Mammograms: 98.6% normal, 1.2% benign, and 0.21% malignant	0.882
GMIC^9^	2021	14,148 Exams from the NYU Breast Cancer Screening dataset v1^16^. Mammograms: 98.6% normal, 1.2% benign, and 0.21% malignant	0.930
DMV-CNN^14^	2020	Reader study on 720 Exams. Mammograms: 43.7% normal, 49.4% benign, and 4.3% malignant	0.886
END2END^13^	2019	A subset of the INbreast database^17^. Mammograms: 107 images from 31 women*	0.950

*We include only the results reported on FFDM images.

Saliency analysis was performed in two steps: (1) generation of the saliency maps and (2) detection of the area of interest by saliency thresholding. For the first step, saliency maps were generated using the Grad-CAM algorithm^18^. Grad-CAM is a well-established method for visualization-based XAI and computes the regions in the input image that yield the highest changes in the final output of the neural network^7^. As a result, it generates a map that assigns a score to each input pixel according to its relevance in the decision-making process. Among the visual explanation methods for XAI, class activation mapping (CAM) methods have been the most widely used in the literature^6^. Gradient-weighted class activation mapping (Grad-CAM) is a generalization of CAM that can be used with any type of CNN to produce post-hoc local explanations, which are a requirement for our study.

**Figure 1:**
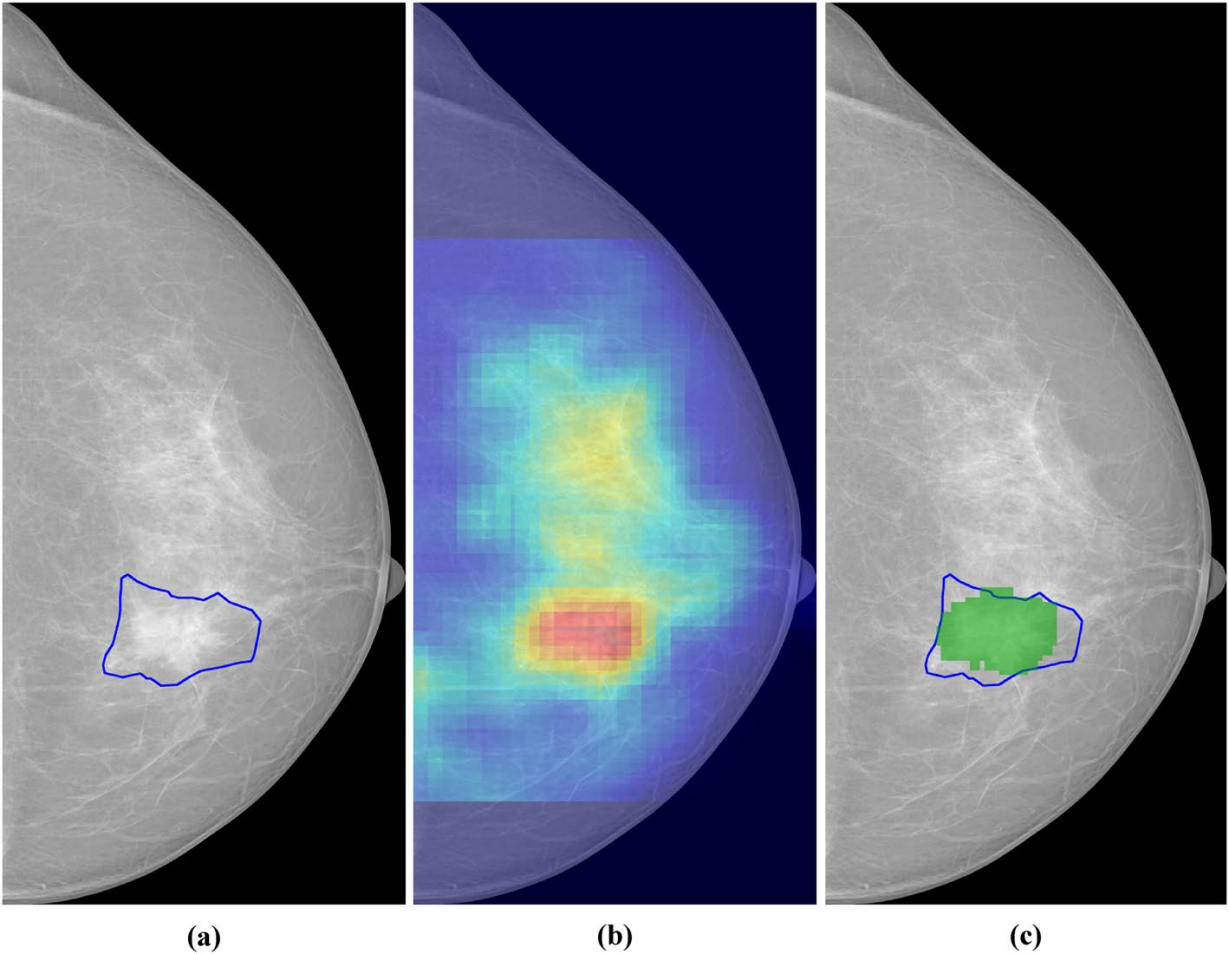
Saliency analysis. (**a**) Mammogram with a manually segmented lesion. (**b**) Saliency map for an AI system. (**c**) Relevant region (in green) obtained by thresholding the saliency map in (**b**).

For the sake of consistency, we normalized all scores between 0 and 1 on a per-image basis, with 1 having the highest saliency. For step (2), we identified the *area of interest* in each mammogram by selecting pixels with a saliency score above a threshold. The saliency threshold was automatically selected through an iterative process that maximizes the overlap between the area of interest and the ground truth of each lesion. This process is illustrated in Fig. 1.

The cancer detection performance of the AI systems was measured using the area under the ROC curve (AUC) with 95% confidence intervals estimated with bootstrapping. Results with *p* < 0*.*05 were considered statistically significant. The AUC is a commonly used measure for assessing the performance of medical diagnosis methods due to its ability to capture the sensitivity–specificity trade-off, robustness to imbalanced data, utility in comparing different tests or models, threshold independence, and interpretability^19^.

The overlap between the areas of interest of each AI system and the breast lesions was measured in terms of the median Dice’s similarity coefficient (DSC) with the interquartile range (IQR). DSC is a widely accepted measure for assessing the quality of segmentation methods due to its sensitivity to spatial overlap, scale invariance, ease of interpretation, and sensitivity to both false positives and false negatives. For each mammogram, the DSC estimates the overlap between a region of interest *A* and a lesion *B* as

1 $$DSC\left(A,B\right)=\frac{2\left|A\cap B\right|}{\left|A\right|\cup \left|B\right|}$$

DSC values range between 0 and 1, with 1 being the total overlap between *A* and *B*. A low DSC value reflects a poor co-localization between the area of interest for an AI system and the delineated breast lesion. In this work, it serves as an effective tool for objective evaluation of the overlap between the areas of interest and manually segmented breast lesions^20,21^.

**Figure 2:**
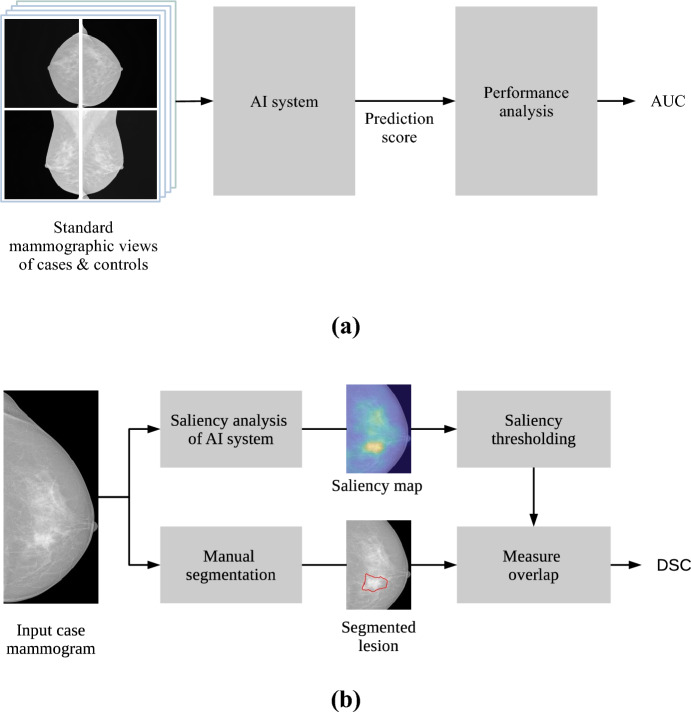
Experimental methodology. (**a**) Analysis of the detection performance of AI systems using the area under the ROC curve (AUC). (**b**) Saliency analysis of breast lesions using Dice’s similarity coefficient (DSC).

The methodology to assess the performance of the AI systems considered in this work is illustrated in Fig. 2a. The methodology for measuring the overlap between breast lesions and the area of interest of AI systems is illustrated in Fig. 2b.

## Results

In the task of cancer detection, the performance of the AI systems was low to moderate. Specifically, three of the systems yielded statistically significant performances with AUCs of 0.569, 0.572, and 0.694 for GMIC, DMV-CNN, and End2End, respectively. The performance of the GLAM method was not statistically significant. The overlap between the area of interest and the lesion in each mammogram was low for all the AI systems, with DSCs between 4.2% and 38.0%. The results are summarized in Table 3.

**Table 3:** Detection performance and saliency analysis.

System	AUC	95% CI	DSC (IQR)
GLAM	0.528	0.494–0.561	0.108 (0.520)
GMIC	0.571	0.535–0.606	0.291 (0.481)
DMV-CNN	0.571	0.536–0.607	0.380 (0.612)
END2END	0.688	0.655–0.719	0.042 (0.151)

## Discussion

### Detection performance of AI systems

Previous research has identified external validation as one of the main difficulties for the adoption of AI systems in screening mammography^22,23^. Our experiments with four state-of-the-art AI systems for screening mammography showed low to moderate performance with our independent, external validation data. Comparison of the AUCs estimated by the original authors of each system (last column of Table 2) and our results (first column of Table 3) shows that the performance of all the systems decreased, with reductions in AUC ranging between 0.256 and 0.361. We argue that this reduction can be attributed to two main factors: differences in test populations and differences in experimental design. We elaborate on the implications of these differences below.

Our test population was different from the populations used in the development of all the AI systems in this study. Breast cancer epidemiology can be significantly affected by the interplay between complex factors, including the population’s mean age, ethnicity, race, lifestyle, environment, socioeconomic status, and healthcare system^24^. To the best of our knowledge, this is the first study to assess the performance of AI systems for cancer detection in screening mammography in Finland. Our results highlight the importance of extensively testing AI systems in populations different from the ones used in the development of the systems.

There were also some important differences in our experimental design that could affect the performance of the AI systems. First, we used a case–control design matched by age and mammographic system. Age is one of the strongest risk factors for breast cancer^25^. In the studies where the AI systems were developed, however, age was not included in the experimental design nor accounted for in the statistical analysis. It is well known that age affects the radiological appearance of breast parenchyma^26^. This, in turn, could affect the performance of the AI systems: the AI systems may have relied at least partly on the age-associated changes, not the breast-related changes. Moreover, previous studies have demonstrated that differences in systems can affect the reliability of computerized mammographic analysis algorithms^27,28^. In fact, recent research has demonstrated the impact of technological settings on the performance of AI systems for breast cancer screening^29^. Finally, a previous history of breast cancer is a strong risk factor^30^. Changes in breast parenchyma due to previous interventions (e.g., metal clips) and treatments (e.g., radiotherapy-associated changes) can be cues for AI systems. Because we excluded symptomatic women and women with previous findings or histories of breast cancer, we believe that our experimental setting represents a more challenging scenario for the detection of breast cancer.

In addition to the aforementioned factors, previous studies have pointed to *overfitting* and *bias* as plausible explanations for the inconsistent performance of AI systems in independent test data^31^. A recent meta-analysis of the external validation of AI systems for screening mammography found that most studies suggest a potential diagnostic improvement when the AI systems are used together with radiologists, but warned about the persistent risk of bias^22^.

### Relevance of breast lesions in cancer detection

In this work, we defined the *area of interest* of an AI system as the region in a mammogram with a saliency level above a threshold. For an input mammogram, this threshold was determined automatically by maximizing the overlap between the area of interest and breast lesions segmented by expert radiologists. Due to the importance of breast lesions in clinical mammographic analysis by radiologists, our hypothesis was that the area of interest of AI systems should have a high overlap with breast lesions. Our results contradict this hypothesis, however. Specifically, the DSCs showed low overlap between regions of interest and breast lesions (median DSC between 4.2% and 38.0%). In addition, the AI system with the highest performance, the End2End system with an AUC of 0.69, showed a remarkably low overlap, with a median DSC of 4.2% (IQR: 15.1%). Our results suggest that, unlike human readers, breast lesions are not as relevant to AI systems when interpreting mammograms. Specifically, the low overlap between the areas of interest and breast lesions suggests that AI systems do not rely on breast lesions as the main decision cue in diagnostics.

**Figure 3:**
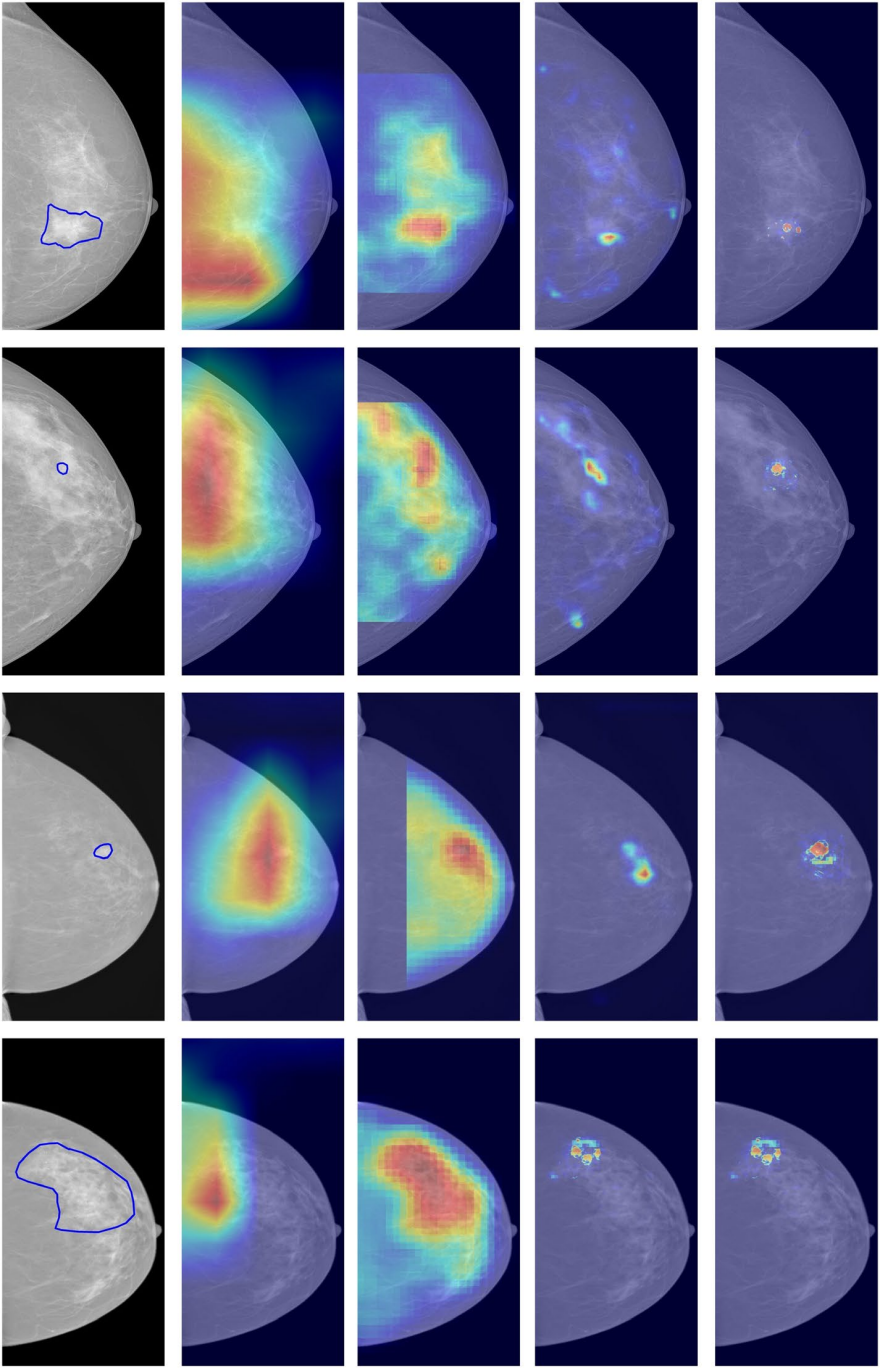
Saliency of AI systems for screening mammograms. From left to right: END2END^13^, DMV-CNN^14^, GMIC^9^, and GLAM^15^. The first two columns show the saliency maps for the best-performing systems in our study. It is clear that saliency shows a high value in a large area within each mammogram, regardless of lesion location and size.

In recent years, the question of the interpretability of AI systems has increasingly gained attention in the machine learning community^3–5,7,32^. In medical imaging, XAI has been identified as one of the key factors in gaining radiologists’ acceptance and, ultimately, fostering its adoption in clinical practice^3^. For the sake of explainability, a highly localized saliency would facilitate the understanding of what image regions or features are more relevant for the AI system. Surprisingly, in our experiments, the highest overlap between the areas of interest and the breast lesions was observed in systems with low detection performance (AUCs between 0.52 and 0.57). As shown in the last two columns of Fig. 3, the systems with the lowest performance, GMIC and GLAM, showed highly localized saliencies. The discussion of these results should take into account the training strategy adopted for the development of the AI system. Among the methods considered in this study, GMIC and GLAM were developed to improve the “interpretability” of the AI system by focusing the analysis on localized regions of interest using labeled data. On the one hand, this helps to explain why the areas of interest of these methods are highly concentrated in specific spatial regions. On the other hand, the lower performance of these methods raises the question of whether the interpretability of AI systems is attained at the expense of detection performance. Our results are highly relevant for the future development of AI systems, as they show that giving a high relevance to breast lesions does not translate into a higher detection performance at the image level.

The finding regarding the relevance of large image regions for the outcome of computerized systems in the analysis of mammograms has been reported before: in breast cancer risk assessment, the extraction of high-throughput quantitative imaging biomarkers in the whole breast region, namely radiomic analysis, has consistently shown promising performance in the prediction of future breast cancer^33^. Based on these findings, some researchers have asked whether small changes in radiological patterns that are inconspicuous to the human eye but occupy large regions in a mammogram could play a role in the detection capabilities of computerized systems^34^. The fact that AI systems use information found in large image regions not circumscribed to lesions is a feasible explanation of why the joint use of AI systems with radiologists outperforms both radiologists and AI systems alone^14,22,35,36^. Our results suggest that successful breast cancer detection using AI systems exploits non-localized image cues not limited to breast lesions.

Our finding has great clinical significance. Recent literature has proposed that stand-alone AI algorithms could, independently or in conjunction with a radiologist, detect breast cancer or triage mammograms. Triaged normal studies could be read in an adapted manner (e.g., by only one reader), and mammograms with suspicious findings could be prioritized^37^. AI systems that detect mammograms with findings suggestive of malignancy, albeit with limited ability to localize the tumor, would be especially beneficial for the triaging purposes of the mammograms. Such algorithms could also potentially replace one of the two readers. Nevertheless, a radiologist would still be needed to confirm the presence of the actual lesions. AI systems that localize the tumors more accurately and yet have worse performance with respect to lesion detection could be used to reduce missed diagnoses. Indeed, the results support the idea that when a developed or to-be-developed AI system is reported, the authors ought to disclose how well the system can detect mammograms with a high likelihood of breast cancer and how well it can localize the lesion.

### Limitations and future work

We identify three main limitations in our work. First, the small sample did not allow for a saliency analysis according to histopathology and tumor grading. Recent works have pointed out how the localization performance of saliency methods changes according to certain tumor-related features, such as the shape and size of lesions^38^. Future research should explore the performance of AI systems while considering clinical information such as breast density, tumor biology, and previous interventions and treatments. This, however, would require a substantially larger sample with annotated lesions. In this regard, we would like to highlight the importance of current efforts in the construction of large screening datasets, including clinical data and image annotations^39–41^.

Another limitation in this study is related to the use of saliency analysis as a means for identifying the regions that most influence the decision-making process of AI systems. Among the existing state-of-the-art methods3^3,6^, we selected a visualization-based method, since we were interested in establishing a connection between the outcome of the AI system and specific imaging features: breast lesions. While interpreting our results, however, one should take into consideration that the interpretability of AI systems remains an open problem, and saliency does not fully explain the decision-making process of AI systems^6^. Few studies have measured how explainability is related to the accuracy of the system. A recent review found that, of 179 works that used XAI, only one reported measures to evaluate the outcome of the XAI^5^. Research on the validity of XAI methods is also scarce^6^. In a recent study^42^, the authors compared four visualization methods for pneumonia detection in chest X-rays: Grad-CAM yielded the best performance. Further investigation of saliency analysis in the context of screening mammography is warranted.

Finally, an unavoidable limitation of our work is the fact that we included a limited number of AI systems that were state-of-the-art. These systems were selected because of their good performance in previous studies and publicly available source code, which enabled the implementation of the saliency analysis. As more AI systems become available, future research is warranted to corroborate our findings.

## Conclusion

We studied the relevance of breast lesions in the decision-making of four AI systems in the mammographic analysis of breast cancer detection. For this purpose, we measured the overlap between the areas of interest in mammograms identified by saliency analysis of the AI systems and the location of breast lesions segmented by expert radiologists. The overlap between the areas of interest and lesion location was low for all four methods, whereas the best-performing methods yielded saliencies that incorporated information from large image regions in the mammogram. Our results suggest that, for the detection of breast cancer, AI systems use image cues not circumscribed to breast lesions.

## Data Availability

The imaging data used in this study is not available due to restrictions on medical data. Source code and generated data are available from the corresponding author on reasonable request.
